# Supplementary material for: A Tool for Rapid Assessment of Functional Outcomes in Patients with Head and Neck Cancer
Source: Cancers (Basel). 2021 Nov 3;13(21):5529. doi: 10.3390/cancers13215529 (PMC8582907; doi:10.3390/cancers13215529)
Supplement: Supplementary file 1 [file cancers-13-05529-s001.zip › cancers-1422330-supplementary.pdf]

Supplementary files

A tool for rapid assessment of functional outcomes in patients with head and neck cancer

Supplemental data S1: Literature search of 120 included articles

Full-text analysis was performed in 68 of 120 included articles, highlighted as bold. Trigger words in the title are underlined.

1: Samuel SR, Maiya AG, Fernandes DJ, Guddattu V, Saxena PUP, Kurian JR, Lin PJ, Mustian KM. Effectiveness of exercise-based rehabilitation on functional capacity and quality of life in head and neck cancer patients receiving chemo-radiotherapy. Support Care Cancer. 2019 Oct;27(10):3913-3920. doi: 10.1007/s00520-019-04750-z. Epub 2019 Mar 27. PMID: 30919154; PMCID: PMC6728220.

2: Grote M, Maihöfer C, Weigl M, Davies-Knorr P, Belka C. Progressive resistance training in cachectic head and neck cancer patients undergoing radiotherapy: a randomized controlled pilot feasibility trial. Radiat Oncol. 2018 Nov 6;13(1):215. doi: 10.1186/s13014-018-1157-0. PMID: 30400971; PMCID: PMC6219249.

3: Mendez A, Seikaly H, Eurich D, Dzioba A, Aalto D, Osswald M, Harris JR, O'Connell DA, Lazarus C, Urken M, Likhterov I, Chai RL, Rauscher E, Buchbinder D, Okay D, Happonen RP, Kinnunen I, Irjala H, Soukka T, Laine J; Head and Neck Research Network. Development of a Patient-Centered Functional Outcomes Questionnaire in Head and Neck Cancer. JAMA Otolaryngol Head Neck Surg. 2020 May 1;146(5):437-443. doi: 10.1001/jamaoto.2019.4788. PMID: 32271362; PMCID: PMC7146527.

4: Capozzi LC, McNeely ML, Lau HY, Reimer RA, Giese-Davis J, Fung TS, Culos-Reed SN. Patient-reported outcomes, body composition, and nutrition status in patients with head and neck cancer: Results from an exploratory randomized controlled exercise trial. Cancer. 2016 Apr 15;122(8):1185-200. doi: 10.1002/cncr.29863. Epub 2016 Feb 1. PMID: 26828426.

5: Nelke KH, Pawlak W, Gerber H, Leszczyszyn J. Head and neck cancer patients' quality of life. Adv Clin Exp Med. 2014 Nov-Dec;23(6):1019-27. doi: 10.17219/acem/37361. PMID: 25618131.

6: Boright L, Doherty DJ, Wilson CM, Arena SK, Ramirez C. Development and Feasibility of a Prehabilitation Protocol for Patients Diagnosed with Head and Neck Cancer. Cureus. 2020 Aug 20;12(8):e9898. doi: 10.7759/cureus.9898. PMID: 32968564; PMCID: PMC7505529.

7: Lin CR, Fan KH, Lin CY, Hung TM, Huang BS, Chen EY, Kang CJ, Huang SF, Chang JT, Chang CH. Development and evaluation of a computerized clinical outcome assessment tool for head and neck cancer patients. Medicine (Baltimore). 2020 Aug 21;99(34):e20304. doi: 10.1097/MD.0000000000020304. PMID: 32846748; PMCID: PMC7447395.

8: van Leeuwen M, Husson O, Alberti P, Arraras JI, Chinot OL, Costantini A, Darlington AS, Dirven L, Eichler M, Hammerlid EB, Holzner B, Johnson CD, Kontogianni M, Kjær TK, Morag O, Nolte S, Nordin A, Pace A, Pinto M, Polz K, Ramage J, Reijneveld JC, Serpentini S, Tomaszewski KA, Vassiliou V, Verdonck-de Leeuw IM, Vistad I, Young TE, Aaronson NK, van de Poll-Franse LV; EORTC QLG. Understanding the quality of life (QOL) issues in survivors of cancer: towards the development of an EORTC QOL cancer survivorship questionnaire. Health Qual Life Outcomes. 2018 Jun 4;16(1):114. doi: 10.1186/s12955-018-0920-0. PMID: 29866185; PMCID: PMC5987570.

9: Zebralla V, Müller J, Wald T, Boehm A, Wichmann G, Berger T, Birnbaum K, Heuermann K, Oeltze-Jafra S, Neumuth T, Singer S, Büttner M, Dietz A, Wiegand S. Obtaining Patient-Reported Outcomes Electronically With "OncoFunction" in Head and Neck Cancer Patients During Aftercare. Front Oncol. 2020 Nov 25;10:549915. doi: 10.3389/fonc.2020.549915. PMID: 33324544; PMCID: PMC7724103.

10: Davudov MM, Rahimov C, Harirchi I, Mirzajani Z, Amiraliyev N, Amiraliyev K, Rustamova N, Zebardast J, Montazeri A. Psychometric evaluation of Azeri version of the head and neck cancer specific quality of life questionnaire (EORTC QLQ-H&N43). Health Qual Life Outcomes. 2020 Jul 23;18(1):248. doi: 10.1186/s12955-020-01500-2. PMID: 32703297; PMCID: PMC7379343.

11: Gelbard A, Anderson C, Berry LD, Amin MR, Benninger MS, Blumin JH, Bock JM, Bryson PC, Castellanos PF, Chen SC, Clary MS, Cohen SM, Crawley BK, Dailey SH, Daniero JJ, de Alarcon A, Donovan DT, Edell ES, Ekbom DC, Fernandes-Taylor S, Fink DS, Franco RA, Garrett CG, Guardiani EA, Hillel AT, Hoffman HT, Hogikyan ND, Howell RJ, Huang LC, Hussain LK, Johns MM 3rd, Kasperbauer JL, Khosla SM, Kinnard C, Kupfer RA, Langerman AJ, Lentz RJ, Lorenz RR, Lott DG, Lowery AS, Makani SS, Maldonado F, Mannion K, Matrka L, McWhorter AJ, Merati AL, Mori MC, Netterville JL, O'Dell K, Ongkasuwan J, Postma GN, Reder LS, Rohde SL, Richardson BE, Rickman OB, Rosen CA, Rutter MJ, Sandhu GS, Schindler JS, Schneider GT, Shah RN, Sikora AG, Sinard RJ, Smith ME, Smith LJ, Soliman AMS, Sveinsdóttir S, Van Daele DJ, Veivers D, Verma SP, Weinberger PM, Weissbrod PA, Wootten CT, Shyr Y, Francis DO. Comparative Treatment Outcomes for Patients With Idiopathic Subglottic Stenosis. JAMA Otolaryngol Head Neck Surg. 2020 Jan 1;146(1):20-29. doi: 10.1001/jamaoto.2019.3022. PMID: 31670805; PMCID: PMC6824232.

12: Rogers SN, Barber B. Using PROMs to guide patients and practitioners through the head and neck cancer journey. Patient Relat Outcome Meas. 2017 Nov 8;8:133-142. doi: 10.2147/PROM.S129012. PMID: 29184455; PMCID: PMC5687779.

13: Alberga JM, Korfage A, Bonnema I, Witjes MJH, Vissink A, Raghoebar GM. Mandibular dental implant placement immediately after teeth removal in head and neck cancer patients. Support Care Cancer. 2020 Dec;28(12):5911-5918. doi: 10.1007/s00520-020-05431-y. Epub 2020 Apr 11. PMID: 32279135; PMCID: PMC7686200.

14: Cmelak A, Dietrich MS, Li S, Ridner S, Forastiere A, Burtness BA, Cella D, Murphy BA. ECOG-ACRIN 2399: analysis of patient related outcomes after Chemoradiation for locally advanced head and neck Cancer. Cancers Head Neck. 2020 Dec 22;5(1):12. doi: 10.1186/s41199-020-00059-1. PMID: 33353553; PMCID: PMC7756946.

15: Jacobi I, van der Molen L, Huiskens H, van Rossum MA, Hilgers FJ. Voice and speech outcomes of chemoradiation for advanced head and neck cancer: a systematic review. Eur Arch Otorhinolaryngol. 2010 Oct;267(10):1495-505. doi: 10.1007/s00405-010-1316-x. Epub 2010 Jun 30. PMID: 20589505; PMCID: PMC2924496.

16: Piai V, Prins JB, Verdonck-de Leeuw IM, Leemans CR, Terhaard CHJ, Langendijk JA, Baatenburg de Jong RJ, Smit JH, Takes RP, Kessels RPC. Assessment of Neurocognitive Impairment and Speech Functioning Before Head and Neck Cancer Treatment. JAMA Otolaryngol Head Neck Surg. 2019 Mar 1;145(3):251-257. doi: 10.1001/jamaoto.2018.3981. PMID: 30676629; PMCID: PMC6439757.

17: Oswald LB, Lee JW, Argiris A, Webster KA, Forastiere AA, Cella D. Validation of brief symptom indexes among patients with recurrent or metastatic squamous cell

carcinoma of the head and neck: A trial of the ECOG-ACRIN Cancer Research Group (E1302). Cancer Med. 2020 Dec;9(23):8884-8894. doi: 10.1002/cam4.3506. Epub 2020 Oct 10. PMID: 33040481; PMCID: PMC7724483.

18: Likhterov I, Ru M, Ganz C, Urken ML, Chai R, Okay D, Liu J, Stewart R, Culliney B, Palacios D, Lazarus CL. Objective and subjective hyposalivation after treatment for head and neck cancer: Long-term outcomes. Laryngoscope. 2018 Dec;128(12):2732-2739. doi: 10.1002/lary.27224. Epub 2018 Oct 16. PMID: 30325025; PMCID: PMC6309704.

19: Ellis MA, Sterba KR, Brennan EA, Maurer S, Hill EG, Day TA, Graboyes EM. A Systematic Review of Patient-Reported Outcome Measures Assessing Body Image Disturbance in Patients with Head and Neck Cancer. Otolaryngol Head Neck Surg. 2019 Jun;160(6):941-954. doi: 10.1177/0194599819829018. Epub 2019 Feb 12. PMID: 30744514; PMCID: PMC6546516.

20: Chera BS, Eisbruch A, Murphy BA, Ridge JA, Gavin P, Reeve BB, Bruner DW, Movsas B. Recommended patient-reported core set of symptoms to measure in head and neck cancer treatment trials. J Natl Cancer Inst. 2014 Jul 8;106(7):dju127. doi: 10.1093/jnci/dju127. PMID: 25006189; PMCID: PMC4192043.

21: Bolt S, Eadie T, Yorkston K, Baylor C, Amtmann D. Variables Associated With Communicative Participation After Head and Neck Cancer. JAMA Otolaryngol Head Neck Surg. 2016 Dec 1;142(12):1145-1151. doi: 10.1001/jamaoto.2016.1198. PMID: 27442853; PMCID: PMC5756625.

22: van Beek FE, Jansen F, Mak L, Lissenberg-Witte BI, Buter J, Vergeer MR, Voortman J, Cuijpers P, Leemans CR, Verdonck-de Leeuw IM. The course of symptoms of anxiety and depression from time of diagnosis up to 2 years follow-up in head and neck cancer patients treated with primary (chemo)radiation. Oral Oncol. 2020 Mar;102:104576. doi: 10.1016/j.oraloncology.2020.104576. Epub 2020 Feb 3. PMID: 32028135.

23: Baudelet M, Van den Steen L, Duprez F, De Bodt M, Deschuymer S, Goeleven A, Hutsebaut I, Mariën S, Meersschout S, Nevens D, Nuyts S, Peeters M, Specenier P, Van den Brekel M, van der Molen L, Vandenbruaene C, Vanderveken O, Van Dinther J, Van Laer C, Vauterin T, Verstraete H, Van Nuffelen G; Member of the Belgian PRESTO Group. Study protocol for a randomized controlled trial: prophylactic swallowing exercises in head-and-neck cancer patients treated with (chemo)radiotherapy (PRESTO trial). Trials. 2020 Mar 2;21(1):237. doi: 10.1186/s13063-020-4171-0. PMID: 32122397; PMCID: PMC7053144.

24: Kamstra JI, Jager-Wittenaar H, Dijkstra PU, Huisman PM, van Oort RP, van der Laan BF, Roodenburg JL. Oral symptoms and functional outcome related to oral and oropharyngeal cancer. Support Care Cancer. 2011 Sep;19(9):1327-33. doi: 10.1007/s00520-010-0952-4. Epub 2010 Aug 13. PMID: 20706851; PMCID: PMC3151373.

25: Sindhar S, Kallogjeri D, Wildes TS, Avidan MS, Piccirillo JF. Association of Preoperative Functional Performance With Outcomes After Surgical Treatment of Head and Neck Cancer: A Clinical Severity Staging System. JAMA Otolaryngol Head Neck Surg. 2019 Dec 1;145(12):1128-1136. doi: 10.1001/jamaoto.2019.1035. PMID: 31045219; PMCID: PMC6499368.

26: Nightingale CL, Sterba KR, Tooze JA, Milliron BJ, Tetrick LA, Paek MS, Weaver KE. Vulnerable characteristics and interest in wellness programs among head and neck cancer caregivers. Support Care Cancer. 2016 Aug;24(8):3437-45. doi: 10.1007/s00520-016-3160-z. Epub 2016 Mar 19. PMID: 26992407; PMCID: PMC4919231.

27: Epstein JB, Wilkie DJ, Fischer DJ, Kim YO, Villines D. Neuropathic and nociceptive pain in head and neck cancer patients receiving radiation therapy. Head Neck Oncol. 2009 Jul 14;1:26. doi: 10.1186/1758-3284-1-26. PMID: 19594943; PMCID: PMC2717963.

28: Verma N, Tan X, Knowles M, Bernard S, Chera B. Patient-reported outcomes for dental health, shoulder-neck dysfunction, and overall quality of life after treatment with radiation for head and neck cancer. Laryngoscope Investig Otolaryngol. 2019 May 2;4(3):300-306. doi: 10.1002/lio2.262. PMID: 31236462; PMCID: PMC6580067.

29: Cracchiolo JR, Klassen AF, Young-Afat DA, Albornoz CR, Cano SJ, Patel SG, Pusic AL, Matros E. Leveraging patient-reported outcomes data to inform oncology clinical decision making: Introducing the FACE-Q Head and Neck Cancer Module. Cancer. 2019 Mar 15;125(6):863-872. doi: 10.1002/cncr.31900. Epub 2018 Nov 30. PMID: 30500993; PMCID: PMC6403001.

30: Verdonck-de Leeuw IM, Jansen F, Brakenhoff RH, Langendijk JA, Takes R, Terhaard CHJ, Baatenburg de Jong RJ, Smit JH, Leemans CR. Advancing interdisciplinary research in head and neck cancer through a multicenter longitudinal prospective cohort study: the NETherlands QUality of life and BIomedical Cohort (NET-QUBIC) data warehouse and biobank. BMC Cancer. 2019 Aug 5;19(1):765. doi: 10.1186/s12885-019-5866-z. Erratum in: BMC Cancer. 2019 Oct 22;19(1):982. PMID: 31382921; PMCID: PMC6683500.

31: Wotherspoon RJ, Kanatas AN, Rogers SN. Review of HaNDLE-on-QoL: a database of published papers that use questionnaires to report quality of life in patients with cancer of the head and neck. Br J Oral Maxillofac Surg. 2018 Feb;56(2):e5-e9. doi: 10.1016/j.bjoms.2017.12.008. Epub 2018 Jan 3. PMID: 29306494.

32: Meyer TK, Pisegna JM, Krisciunas GP, Pauloski BR, Langmore SE. Residue influences quality of life independently of penetration and aspiration in head and neck cancer survivors. Laryngoscope. 2017 Jul;127(7):1615-1621. doi: 10.1002/lary.26387. Epub 2016 Nov 9. PMID: 27861932; PMCID: PMC5423863.

33: Silveira AP, Gonçalves J, Sequeira T, Ribeiro C, Lopes C, Monteiro E, Pimentel FL. Patient reported outcomes in head and neck cancer: selecting instruments for quality of life integration in clinical protocols. Head Neck Oncol. 2010 Oct 31;2:32. doi: 10.1186/1758-3284-2-32. PMID: 21040524; PMCID: PMC2988779.

34: Epstein JB, Beaumont JL, Gwede CK, Murphy B, Garden AS, Meredith R, Le QT, Brizel D, Isitt J, Cella D. Longitudinal evaluation of the oral mucositis weekly questionnaire-head and neck cancer, a patient-reported outcomes questionnaire. Cancer. 2007 May 1;109(9):1914-22. doi: 10.1002/cncr.22620. PMID: 17377917.

35: Duman-Lubberding S, van Uden-Kraan CF, Jansen F, Witte BI, Eerenstein SEJ, van Weert S, de Bree R, Leemans CR, Verdonck-de Leeuw IM. Durable usage of patient-reported outcome measures in clinical practice to monitor health-related quality of life in head and neck cancer patients. Support Care Cancer. 2017 Dec;25(12):3775-

3783. doi: 10.1007/s00520-017-3808-3. Epub 2017 Jul 12. PMID: 28702685; PMCID: PMC5658458.

36: Jackson LK, Ridner SH, Deng J, Bartow C, Mannion K, Niermann K, Gilbert J, Dietrich MS, Cmelak AJ, Murphy BA. Internal Lymphedema Correlates with Subjective and Objective Measures of Dysphagia in Head and Neck Cancer Patients. J Palliat Med. 2016 Sep;19(9):949-56. doi: 10.1089/jpm.2016.0018. Epub 2016 May 26. PMID: 27227341; PMCID: PMC5011629.

37: Rinkel RN, Verdonck-de Leeuw IM, Doornaert P, Buter J, de Bree R, Langendijk JA, Aaronson NK, Leemans CR. Prevalence of swallowing and speech problems in daily life after chemoradiation for head and neck cancer based on cut-off scores of the patient-reported outcome measures SWAL-QOL and SHI. Eur Arch Otorhinolaryngol. 2016 Jul;273(7):1849-55. doi: 10.1007/s00405-015-3680-z. Epub 2015 Jun 14. PMID: 26071622; PMCID: PMC4899484.

38: Scharloo M, Baatenburg de Jong RJ, Langeveld TP, van Velzen-Verkaik E, Doorn-Op den Akker MM, Kaptein AA. Illness cognitions in head and neck squamous cell carcinoma: predicting quality of life outcome. Support Care Cancer. 2010 Sep;18(9):1137-45. doi: 10.1007/s00520-009-0728-x. Epub 2009 Aug 29. PMID: 19718524; PMCID: PMC2910308.

39: Nogueira TE, Adorno M, Mendonça E, Leles C. Factors associated with the quality of life of subjects with facial disfigurement due to surgical treatment of head and neck cancer. Med Oral Patol Oral Cir Bucal. 2018 Mar 1;23(2):e132-e137. doi: 10.4317/medoral.22072. PMID: 29476675; PMCID: PMC5911363.

40: Barber B, Dergousoff J, Nesbitt M, Mitchell N, Harris J, O'Connell D, Côté D, Biron V, Seikaly H. Depression as a predictor of postoperative functional performance status (PFPS) and treatment adherence in head and neck cancer patients: a prospective study. J Otolaryngol Head Neck Surg. 2015 Sep 18;44(1):38. doi: 10.1186/s40463-015-0092-4. PMID: 26385356; PMCID: PMC4574730.

41: Hansson E, Carlström E, Olsson LE, Nyman J, Koinberg I. Can a person-centred-care intervention improve health-related quality of life in patients with head and neck cancer? A randomized, controlled study. BMC Nurs. 2017 Feb 21;16:9. doi: 10.1186/s12912-017-0206-6. PMID: 28239295; PMCID: PMC5320778.

42: Pearman TP, Beaumont JL, Paul D, Abernethy AP, Jacobsen PB, Syrjala KL, Von Roenn J, Cella D. Evaluation of treatment- and disease-related symptoms in advanced head and neck cancer: validation of the national comprehensive cancer network-functional assessment of cancer therapy-head and neck cancer symptom index-22 (NFHNSI-22). J Pain Symptom Manage. 2013 Jul;46(1):113-20. doi: 10.1016/j.jpainsymman.2012.06.004. Epub 2012 Sep 25. PMID: 23017622; PMCID: PMC5886783.

43: Dzioba A, Aalto D, Papadopoulos-Nydam G, Seikaly H, Rieger J, Wolfaardt J, Osswald M, Harris JR, O'Connell DA, Lazarus C, Urken M, Likhterov I, Chai RL, Rauscher E, Buchbinder D, Okay D, Happonen RP, Kinnunen I, Irjala H, Soukka T, Laine J; Head and Neck Research Network. Functional and quality of life outcomes after partial glossectomy: a multi-institutional longitudinal study of the head and neck research network. J Otolaryngol Head Neck Surg. 2017 Sep 4;46(1):56. doi: 10.1186/s40463-017-0234-y. Erratum in: J Otolaryngol Head Neck Surg. 2017 Oct 5;46(1):59. PMID: 28870248; PMCID: PMC5583999.

44: Kwon M, Kim SA, Roh JL, Lee SW, Kim SB, Choi SH, Nam SY, Kim SY. An Introduction to a Head and Neck Cancer-Specific Frailty Index and Its Clinical Implications in Elderly Patients: A Prospective Observational Study Focusing on Respiratory and Swallowing Functions. Oncologist. 2016 Sep;21(9):1091-8. doi: 10.1634/theoncologist.2016-0008. Epub 2016 Jul 1. PMID: 27368883; PMCID: PMC5016072.

45: Gobbo M, Bullo F, Perinetti G, Gatto A, Ottaviani G, Biasotto M, Tirelli G. Diagnostic and therapeutic features associated with modification of quality-of-life's outcomes between one and six months after major surgery for head and neck cancer. Braz J Otorhinolaryngol. 2016 Sep-Oct;82(5):548-57. doi: 10.1016/j.bjorl.2015.10.013. Epub 2015 Dec 18. PMID: 26878840.

46: Patterson JM, Fay M, Exley C, McColl E, Breckons M, Deary V. Feasibility and acceptability of combining cognitive behavioural therapy techniques with swallowing therapy in head and neck cancer dysphagia. BMC Cancer. 2018 Jan 2;18(1):1. doi: 10.1186/s12885-017-3892-2. PMID: 29291726; PMCID: PMC5748941.

47: Eickmeyer SM, Walczak CK, Myers KB, Lindstrom DR, Layde P, Campbell BH. Quality of life, shoulder range of motion, and spinal accessory nerve status in 5-year survivors of head and neck cancer. PM R. 2014 Dec;6(12):1073-80. doi: 10.1016/j.pmrj.2014.05.015. Epub 2014 May 28. PMID: 24880060; PMCID: PMC4247358.

48: Fingeret MC, Hutcheson KA, Jensen K, Yuan Y, Urbauer D, Lewin JS. Associations among speech, eating, and body image concerns for surgical patients with head and neck cancer. Head Neck. 2013 Mar;35(3):354-60. doi: 10.1002/hed.22980. Epub 2012 Mar 20. PMID: 22431304; PMCID: PMC4022133.

49: Barroso EM, Carvalho AL, Paiva CE, Murphy BA, Paiva BS. The Vanderbilt Head and Neck Symptom Survey Brazilian Portuguese version 2.0 (VHNSS 2.0): psychometric properties for patients with head and neck cancer who have undergone radiotherapy. BMC Res Notes. 2015 Oct 1;8:522. doi: 10.1186/s13104-015-1470-8. PMID: 26428472; PMCID: PMC4589957.

50: Wan Leung S, Lee TF, Chien CY, Chao PJ, Tsai WL, Fang FM. Health-related quality of life in 640 head and neck cancer survivors after radiotherapy using EORTC QLQ-C30 and QLQ-H&N35 questionnaires. BMC Cancer. 2011 Apr 12;11:128. doi: 10.1186/1471-2407-11-128. PMID: 21486431; PMCID: PMC3083374.

51: Jansen F, Witte BI, van Uden-Kraan CF, Braspenning AM, Leemans CR, Verdonck- de Leeuw IM. The need for supportive care among head and neck cancer patients: psychometric assessment of the Dutch version of the Supportive Care Needs Survey Short-Form (SCNS-SF34) and the newly developed head and neck cancer module (SCNS-HNC). Support Care Cancer. 2016 Nov;24(11):4639-49. doi: 10.1007/s00520-016-3307-y. Epub 2016 Jun 18. PMID: 27318479; PMCID: PMC5031728.

52: Elting LS, Keefe DM, Sonis ST, Garden AS, Spijkervet FK, Barasch A, Tishler RB, Canty TP, Kudrimoti MK, Vera-Llonch M; Burden of Illness Head and Neck Writing Committee. Patient-reported measurements of oral mucositis in head and neck cancer patients treated with radiotherapy with or without chemotherapy: demonstration of increased frequency, severity, resistance to palliation, and impact on quality of life. Cancer. 2008 Nov 15;113(10):2704-13. doi: 10.1002/cncr.23898. PMID: 18973181.

53: MD Anderson Head and Neck Cancer Symptom Working Group, Kamal M, Rosenthal DI, Volpe S, Goepfert RP, Garden AS, Hutcheson KA, Al Feghali KA, Meheissen MAM, Eraj SA, Dursteler AE, Williams B, Smith JB, Aymard JM, Berends J, White AL, Frank SJ, Morrison WH, Cardoso R, Chambers MS, Sturgis EM, Mendoza TR, Lu C, Mohamed ASR, Fuller CD, Gunn GB. Patient reported dry mouth: Instrument comparison and model performance for correlation with quality of life in head and neck cancer survivors. Radiother Oncol. 2018 Jan;126(1):75-80. doi: 10.1016/j.radonc.2017.10.037. Epub 2017 Dec 8. PMID: 29229507; PMCID: PMC5957088.

54: Hodder SC, Edwards MJ, Brickley MR, Shepherd JP. Multiattribute utility assessment of outcomes of treatment for head and neck cancer. Br J Cancer. 1997;75(6):898-902. doi: 10.1038/bjc.1997.158. PMID: 9062413; PMCID: PMC2063410.

55: van Herpen CM, Mauer ME, Mesia R, Degardin M, Jelic S, Coens C, Betka J, Bernier J, Remenar E, Stewart JS, Preiss JH, van den Weyngaert D, Bottomley A, Vermorken JB; EORTC Head and Neck Group. Short-term health-related quality of life and symptom control with docetaxel, cisplatin, 5-fluorouracil and cisplatin (TPF), 5-fluorouracil (PF) for induction in unresectable locoregionally advanced head and neck cancer patients (EORTC 24971/TAX 323). Br J Cancer. 2010 Oct 12;103(8):1173-81. doi: 10.1038/sj.bjc.6605860. Epub 2010 Sep 14. PMID: 20842129; PMCID: PMC2967049.

56: Yadav K, Nair D, Agarwal J, Chakrabarti S, Joshi S, Ingole P, Mishra A, Tathe N, Chaturvedi P. Prevalence of Functional Problems After Oral Cavity Malignancy Treatment at a Tertiary Center: Utilizing PSS HN (Performance Status Scale for Head and Neck) Scale. J Maxillofac Oral Surg. 2016 Mar;15(1):38-44. doi: 10.1007/s12663-015-0805-2. Epub 2015 Jun 4. PMID: 26929551; PMCID: PMC4759033.

57: Yang CJ, Roh JL, Choi KH, Kim MJ, Choi SH, Nam SY, Kim SY. Pretreatment Dysphagia Inventory and videofluorographic swallowing study as prognostic indicators of early survival outcomes in head and neck cancer. Cancer. 2015 May 15;121(10):1588-98. doi: 10.1002/cncr.29245. Epub 2015 Jan 13. PMID: 25639759.

58: Tykodi SS, Schadendorf D, Cella D, Reck M, Harrington K, Wagner S, Shaw JW. Patient-reported outcomes with nivolumab in advanced solid cancers. Cancer Treat Rev. 2018 Nov;70:75-87. doi: 10.1016/j.ctrv.2018.08.001. Epub 2018 Aug 2. PMID: 30125799.

59: Kenzik KM, Kent EE, Martin MY, Bhatia S, Pisu M. Chronic condition clusters and functional impairment in older cancer survivors: a population-based study. J Cancer Surviv. 2016 Dec;10(6):1096-1103. doi: 10.1007/s11764-016-0553-4. Epub 2016 May 27. PMID: 27229869; PMCID: PMC5096973.

60: McNeely ML, Parliament MB, Seikaly H, Jha N, Magee DJ, Haykowsky MJ, Courneya KS. Sustainability of outcomes after a randomized crossover trial of resistance exercise for shoulder dysfunction in survivors of head and neck cancer. Physiother Can. 2015 Winter;67(1):85-93. doi: 10.3138/ptc.2014-13O. PMID: 25931658; PMCID: PMC4403331.

61: Spiegel JL, Pilavakis Y, Canis M, Welz C. Shoulder Morbidity in Patients after Head and Neck Reconstruction with the Pedicled Supraclavicular Island Flap.

Plast Reconstr Surg Glob Open. 2018 Apr 12;6(4):e1711. doi: 10.1097/GOX.0000000000001711. PMID: 29876164; PMCID: PMC5977956.

62: Giordano L, Sarandria D, Fabiano B, Del Carro U, Bussi M. Shoulder function after selective and superselective neck dissections: clinical and functional outcomes. Acta Otorhinolaryngol Ital. 2012 Dec;32(6):376-9. PMID: 23349556; PMCID: PMC3552537.

63: Şencan Z, Cömert E, Tunçel Ü, Kılıç C. Voice and Quality-of-Life Outcomes of Diode Laser for Tis-T1a Glottic Cancer. Ear Nose Throat J. 2020 May;99(4):229-234. doi: 10.1177/0145561319839868. Epub 2019 Apr 23. PMID: 31012346.

64: Zica GM, Freitas AS, Silva ACAE, Dias FL, Santos IC, Freitas EQ, Koch HA. Swallowing, voice and quality of life of patients submitted to extended supratracheal laryngectomy. Einstein (Sao Paulo). 2020;18:eAO5390. doi: 10.31744/einstein_journal/2020ao5390. Epub 2020 May 18. PMID: 32428067; PMCID: PMC7233281.

65: Braam PM, Roesink JM, Raaijmakers CP, Busschers WB, Terhaard CH. Quality of life and salivary output in patients with head-and-neck cancer five years after radiotherapy. Radiat Oncol. 2007 Jan 5;2:3. doi: 10.1186/1748-717X-2-3. PMID: 17207274; PMCID: PMC1779273.

66: Cohen WA, Albornoz CR, Cordeiro PG, Cracchiolo J, Encarnacion E, Lee M, Cavalli M, Patel S, Pusic AL, Matros E. Health-Related Quality of Life following Reconstruction for Common Head and Neck Surgical Defects. Plast Reconstr Surg. 2016 Dec;138(6):1312-1320. doi: 10.1097/PRS.0000000000002766. PMID: 27879602; PMCID: PMC5559272.

67: Borggreven PA, Verdonck-de Leeuw IM, Muller MJ, Heiligers ML, de Bree R, Aaronson NK, Leemans CR. Quality of life and functional status in patients with cancer of the oral cavity and oropharynx: pretreatment values of a prospective study. Eur Arch Otorhinolaryngol. 2007 Jun;264(6):651-7. doi: 10.1007/s00405-007-0249-5. Epub 2007 Feb 2. PMID: 17273840; PMCID: PMC1914238.

68: Petrovic I, Baser R, Blackwell T, McCarthy C, Ganly I, Patel S, Cordeiro P, Shah J. Long-term functional and esthetic outcomes after fibula free flap reconstruction of the mandible. Head Neck. 2019 Jul;41(7):2123-2132. doi: 10.1002/hed.25666. Epub 2019 Feb 13. PMID: 30761650; PMCID: PMC6579701.

69: Cocuzza S, Maniaci A, Grillo C, Ferlito S, Spinato G, Coco S, Merlino F, Stilo G, Santoro GP, Iannella G, Vicini C, La Mantia I. Voice-Related Quality of Life in Post-Laryngectomy Rehabilitation: Tracheoesophageal Fistula's Wellness. Int J Environ Res Public Health. 2020 Jun 26;17(12):4605. doi: 10.3390/ijerph17124605. PMID: 32604875; PMCID: PMC7344397.

70: Vella O, Blanchard D, de Raucourt D, Rame JP, Babin E. Function evaluation of laryngeal reconstruction using infrahyoid muscle after partial laryngectomy in 37 patients. Eur Ann Otorhinolaryngol Head Neck Dis. 2020 Jan;137(1):7-11. doi: 10.1016/j.anorl.2019.09.004. Epub 2019 Sep 17. PMID: 31540850.

71: Onakoya PA, Nwaorgu OG, Adenipekun AO, Aluko AA, Ibekwe TS. Quality of life in patients with head and neck cancers. J Natl Med Assoc. 2006 May;98(5):765-70. PMID: 16749653; PMCID: PMC2569276.

72: Tuomi L, Karlsson T. Voice Quality, Function, and Quality of Life for Laryngeal Cancer: A Prospective Longitudinal Study Up to 24 Months Following Radiotherapy. Ear Nose Throat J. 2020 Jun 2:145561320929941. doi: 10.1177/0145561320929941. Epub ahead of print. PMID: 32484410.

73: Del Bon F, Piazza C, Mangili S, Redaelli De Zinis LO, Nicolai P, Peretti G. Transoral laser surgery for recurrent glottic cancer after radiotherapy: oncologic and functional outcomes. Acta Otorhinolaryngol Ital. 2012 Aug;32(4):229-37. PMID: 23093812; PMCID: PMC3468937.

74: Scott SI, Kathrine Ø Madsen A, Rubek N, Charabi BW, Wessel I, Fredslund Hadjú S, Jensen CV, Stephen S, Patterson JM, Friborg J, Hutcheson KA, Kehlet H, von Buchwald C. Long-term quality of life & functional outcomes after treatment of oropharyngeal cancer. Cancer Med. 2021 Jan;10(2):483-495. doi: 10.1002/cam4.3599. Epub 2020 Dec 4. PMID: 33277795; PMCID: PMC7877346.

75: Macefield RC, Jacobs M, Korfage IJ, Nicklin J, Whistance RN, Brookes ST, Sprangers MA, Blazeby JM. Developing core outcomes sets: methods for identifying and including patient-reported outcomes (PROs). Trials. 2014 Feb 5;15:49. doi: 10.1186/1745-6215-15-49. PMID: 24495582; PMCID: PMC3916696.

76: Hendriksma M, van Loon Y, Klop WMC, Hakkesteegt MM, Heijnen BJ, El Hasnaoui I, de Jong M, Langeveld TPM, van Benthem PPG, Baatenburg de Jong RJ, Sjögren EV. Quality of life and voice outcome of patients treated with transoral CO2 laser microsurgery for early glottic carcinoma (T1-T2): a 2-year follow-up study. Eur Arch Otorhinolaryngol. 2019 Mar;276(3):805-814. doi: 10.1007/s00405-019-05348-1. Epub 2019 Feb 27. PMID: 30810819; PMCID: PMC6411677.

77: Spellun AH, Moreland CJ, Kushalnagar P. Young Deaf Adults' Knowledge of Human Papillomavirus and Human Papillomavirus Vaccine's Effectiveness in Preventing Cervical, Anal, Penile, and Oral Cancer. J Pediatr Adolesc Gynecol. 2019 Jun;32(3):293-299. doi: 10.1016/j.jpag.2018.11.013. Epub 2018 Dec 7. PMID: 30529699; PMCID: PMC6555690.

78: Chepeha DB, Taylor RJ, Chepeha JC, Teknos TN, Bradford CR, Sharma PK, Terrell JE, Wolf GT. Functional assessment using Constant's Shoulder Scale after modified radical and selective neck dissection. Head Neck. 2002 May;24(5):432-6. doi: 10.1002/hed.10067. PMID: 12001072.

79: Bui AT, Yong Ji KS, Pham CT, Le KM, Tong TX, Lee WT. Longitudinal evaluation of quality of life in Laryngeal Cancer patients treated with surgery. Int J Surg. 2018 Oct;58:65-70. doi: 10.1016/j.ijsu.2018.09.011. Epub 2018 Sep 24.
PMID: 30261330.

80: Rettig EM, D'Souza G, Thompson CB, Koch WM, Eisele DW, Fakhry C. Health-related quality of life before and after head and neck squamous cell carcinoma: Analysis of the Surveillance, Epidemiology, and End Results-Medicare Health Outcomes Survey linkage. Cancer. 2016 Jun 15;122(12):1861-70. doi: 10.1002/cncr.30005. Epub 2016 May 16. PMID: 27182694; PMCID: PMC4950667.

81: Hurtuk AM, Marcinow A, Agrawal A, Old M, Teknos TN, Ozer E. Quality-of-life outcomes in transoral robotic surgery. Otolaryngol Head Neck Surg. 2012 Jan;146(1):68-73. doi: 10.1177/0194599811421298. Epub 2011 Aug 31. PMID: 21881053; PMCID: PMC4277658.

82: Jehn P, Stier R, Tavassol F, Dittmann J, Zimmerer R, Gellrich NC, Krüskemper G, Spalthoff S. Physical and Psychological Impairments Associated with Mucositis after Oral Cancer Treatment and Their Impact on Quality of Life. Oncol Res Treat. 2019;42(6):342-349. doi: 10.1159/000499720. Epub 2019 Apr 10. PMID: 30970370.

83: Souza FGR, Santos IC, Bergmann A, Thuler LCS, Freitas AS, Freitas EQ, Dias FL. Quality of life after total laryngectomy: impact of different vocal rehabilitation methods in a middle income country. Health Qual Life Outcomes. 2020 Apr 3;18(1):92. doi: 10.1186/s12955-020-1281-z. PMID: 32245483; PMCID: PMC7126368.

84: Sakthivel P, K Irugu DV, Singh CA, Verma H, Yogal R, Jat B, Chadran A, Sikka K, Thakar A, Sharma SC. Quality of life outcome measures using University of Washington questionnaire version 4 in early T1/T2 anterior tongue cancers with and without radiotherapy: A cross-sectional study. Indian J Cancer. 2017 Apr-Jun;54(2):447-452. doi: 10.4103/ijc.IJC_236_17. PMID: 29469076.

85: Chapuy CI, Annino DJ, Snavely A, Li Y, Tishler RB, Norris CM, Haddad RI, Goguen LA. Swallowing function following postchemoradiotherapy neck dissection: review of findings and analysis of contributing factors. Otolaryngol Head Neck Surg. 2011 Sep;145(3):428-34. doi: 10.1177/0194599811403075. PMID: 21493276; PMCID: PMC3434459.

86: Hansen CC, Smith JB, Mohamed ASR, Mulcahy CF, Wefel JS, Hutcheson KA, Chrane K, Phan J, Frank SJ, Garden AS, Smith BD, Eichelberger H, Anderson C, McCoy C, Horiates M, Patrick C, Floris S, French C, Beadle BM, Morrison WH, Su SY, Lewis CM, Kupferman ME, Johnson JM, Skinner HD, Lai SY, Hanna EY, Rosenthal DI, Fuller CD, Gunn GB; MD Anderson Head and Neck Cancer Symptom Working Group. Cognitive function and patient-reported memory problems after radiotherapy for cancers at the skull base: A cross-sectional survivorship study using the Telephone Interview for Cognitive Status and the MD Anderson Symptom Inventory-Head and Neck Module. Head Neck. 2017 Oct;39(10):2048-2056. doi: 10.1002/hed.24876. Epub 2017 Aug 1. PMID: 28763137; PMCID: PMC6082378.

87: Keereweer S, de Wilt JH, Sewnaik A, Meeuwis CA, Tilanus HW, Kerrebijn JD. Early and long-term morbidity after total laryngopharyngectomy. Eur Arch Otorhinolaryngol. 2010 Sep;267(9):1437-44. doi: 10.1007/s00405-010-1244-9. Epub 2010 Apr 7. PMID: 20372916; PMCID: PMC2908431.

88: Reddy GRK, Hulikal N, Lakshmi AY, Vengamma B. Nerve and vein preserving neck dissections for oral cancers: a prospective evaluation of spinal accessory nerve function and internal jugular vein patency following treatment. Acta Otorhinolaryngol Ital. 2018 Feb;38(1):7-12. doi: 10.14639/0392-100X-1481. Epub 2017 Oct 31. PMID: 29087403; PMCID: PMC5952988.

89: Hegde JV, Shaverdian N, Daly ME, Felix C, Wong DL, Rosove MH, Garst JH, Wang PC, Veruttipong D, Rao S, Fragoso RC, Riess JW, Steinberg ML, Chen AM. Patient- reported quality-of-life outcomes after de-escalated chemoradiation for human papillomavirus-positive oropharyngeal carcinoma: Findings from a phase 2 trial. Cancer. 2018 Feb 1;124(3):521-529. doi: 10.1002/cncr.30954. Epub 2017 Oct 17. PMID: 29044458; PMCID: PMC5916816.

90: Feng FY, Kim HM, Lyden TH, Haxer MJ, Worden FP, Feng M, Moyer JS, Prince ME, Carey TE, Wolf GT, Bradford CR, Chepeha DB, Eisbruch A. Intensity-modulated chemoradiotherapy aiming to reduce dysphagia in patients with oropharyngeal cancer: clinical and functional results. J Clin Oncol. 2010 Jun 1;28(16):2732-8. doi: 10.1200/JCO.2009.24.6199. Epub 2010 Apr 26. PMID: 20421546; PMCID: PMC2881852.

91: Petrovic I, Panchal H, De Souza Franca PD, Hernandez M, McCarthy CC, Shah JP. A systematic review of validated tools assessing functional and aesthetic outcomes following fibula free flap reconstruction of the mandible. Head Neck. 2019 Jan;41(1):248-255. doi: 10.1002/hed.25452. Epub 2018 Dec 12. PMID: 30548509; PMCID: PMC6339585.

92: Judy GD, Green R, Aumer SL, Amdur RJ, Tan X, Sheets N, Weissler M, Zanation A, Patel S, Hackman T, Mendenhall WM, Chera BS. Preservation of swallowing function with de-intensified chemoradiation therapy for HPV-associated oropharyngeal squamous cell carcinoma. Adv Radiat Oncol. 2018 Mar 14;3(3):356-365. doi: 10.1016/j.adro.2018.03.002. PMID: 30197941; PMCID: PMC6127973.

93: Petersen MA, Aaronson NK, Arraras JI, Chie WC, Conroy T, Costantini A, Dirven L, Fayers P, Gamper EM, Giesinger JM, Habets EJJ, Hammerlid E, Helbostad J, Hjermstad MJ, Holzner B, Johnson C, Kemmler G, King MT, Kaasa S, Loge JH, Reijneveld JC, Singer S, Taphoorn MJB, Thamsborg LH, Tomaszewski KA, Velikova G, Verdonck-de Leeuw IM, Young T, Groenvold M; European Organisation for Research and Treatment of Cancer (EORTC) Quality of Life Group. The EORTC CAT Core-The computer adaptive version of the EORTC QLQ-C30 questionnaire. Eur J Cancer. 2018 Sep;100:8-16. doi: 10.1016/j.ejca.2018.04.016. Epub 2018 Jun 21. PMID: 29936066.

94: Strieth S, Ernst BP, Both I, Hirth D, Pfisterer LN, Künzel J, Eder K. Randomized controlled single-blinded clinical trial of functional voice outcome after vascular targeting KTP laser microsurgery of early laryngeal cancer. Head Neck. 2019 Apr;41(4):899-907. doi: 10.1002/hed.25474. Epub 2019 Jan 31. PMID: 30702173; PMCID: PMC6972647.

95: Wyatt G, Pugh SL, Wong RK, Sagar S, Singh AK, Koyfman SA, Nguyen-Tân PF, Yom SS, Cardinale FS, Sultanem K, Hodson I, Krempl GA, Lukaszczyk B, Yeh AM, Berk L. Xerostomia health-related quality of life: NRG oncology RTOG 0537. Qual Life Res. 2016 Sep;25(9):2323-33. doi: 10.1007/s11136-016-1255-0. Epub 2016 Feb 25. PMID: 26914104; PMCID: PMC4982852.

96: Tsai WL, Huang TL, Liao KC, Chuang HC, Lin YT, Lee TF, Huang HY, Fang FM. Impact of late toxicities on quality of life for survivors of nasopharyngeal carcinoma. BMC Cancer. 2014 Nov 21;14:856. doi: 10.1186/1471-2407-14-856. PMID: 25413127; PMCID: PMC4247772.

97: Barrios R, Bravo M, Gil-Montoya JA, Martínez-Lara I, García-Medina B, Tsakos G. Oral and general health-related quality of life in patients treated for oral cancer compared to control group. Health Qual Life Outcomes. 2015 Jan 23;13:9. doi: 10.1186/s12955-014-0201-5. PMID: 25613348; PMCID: PMC4311475.

98: de Groot RJ, Rosenberg AJWP, van der Bilt A, Aalto D, Merkx MAW, Speksnijder CM. The association between a mixing ability test and patient reported chewing ability in patients treated for oral malignancies. J Oral Rehabil. 2019

Feb;46(2):140-150. doi: 10.1111/joor.12734. Epub 2018 Nov 1. PMID: 30311256; PMCID: PMC7379969.

99: Williamson JS, Ingrams D, Jones H. Quality of life after treatment of laryngeal carcinoma: a single centre cross-sectional study. Ann R Coll Surg Engl. 2011 Nov;93(8):591-5. doi: 10.1308/147870811X13137608455253. PMID: 22041234; PMCID: PMC3566683.

100: Yang H, Han D, Ren X, Luo H, Li X. Investigation of swallowing function and <u>swallowing</u>-related quality of life after partial laryngectomy in Chinese patients with laryngeal carcinoma. Health Qual Life Outcomes. 2019 Jul 26;17(1):132. doi: 10.1186/s12955-019-1199-5. PMID: 31349839; PMCID: PMC6660951.

101: Ringash J, O'Sullivan B, Bezjak A, Redelmeier DA. Interpreting clinically significant changes in patient-reported outcomes. Cancer. 2007 Jul 1;110(1):196-202. doi: 10.1002/cncr.22799. PMID: 17546575.

102: Buurman DJM, Speksnijder CM, Engelen BHBT, Kessler P. <u>Masticatory performance</u> and oral health-related quality of life in edentulous maxillectomy patients: A cross-sectional study to compare implant-supported obturators and conventional obturators. Clin Oral Implants Res. 2020 May;31(5):405-416. doi: 10.1111/clr.13577. Epub 2020 Jan 27. PMID: 31944417; PMCID: PMC7319476.

103: Stomeo F, Tosin E, Morolli F, Bianchini C, Ciorba A, Pastore A, Pelucchi S. Comparison of subjective and objective tools in transoral laser cordectomy for early glottic cancer: importance of <u>voice</u> handicap index. Int J Immunopathol Pharmacol. 2013 Apr-Jun;26(2):445-51. doi: 10.1177/039463201302600217. PMID: 23755759.

104: Soares JRN, Dias FL, Lima RRMA, Toscano UB, Pontes ACP, Botinelly RD, Souza FGR, Araujo Filho VJF, Matos LL, Cernea CR. Assessment of quality of life in patients with advanced oral cancer who underwent mandibulectomy with or without bone reconstruction. Rev Assoc Med Bras (1992). 2018 Aug;64(8):710-716. doi: 10.1590/1806-9282.64.08.710. PMID: 30673041.

105: Szuecs M, Kuhnt T, Punke C, Witt G, Klautke G, Kramp B, Hildebrandt G. Subjective <u>voice</u> quality, <u>communicative ability</u> and <u>swallowing</u> after definitive radio(chemo)therapy, laryngectomy plus radio(chemo)therapy, or organ conservation surgery plus radio(chemo)therapy for laryngeal and hypopharyngeal cancer. J Radiat Res. 2015 Jan;56(1):159-68. doi: 10.1093/jrr/rru093. Epub 2014 Oct 26. PMID: 25348250; PMCID: PMC4572584.

106: Aggarwal P, Zaveri JS, Goepfert RP, Shi Q, Du XL, Swartz M, Lai SY, Fuller CD, Lewin JS, Piller LB, Hutcheson KA. <u>Swallowing</u>-related outcomes associated with late lower cranial neuropathy in long-term oropharyngeal cancer survivors: cross-sectional survey analysis. Head Neck. 2019 Nov;41(11):3880-3894. doi: 10.1002/hed.25923. Epub 2019 Aug 23. PMID: 31441572; PMCID: PMC7240804.

107: Goepfert RP, Lewin JS, Barrow MP, Fuller CD, Lai SY, Song J, Hobbs BP, Gunn GB, Beadle BM, Rosenthal DI, Garden AS, Kies MS, Papadimitrakopoulou VA, Schwartz DL, Hutcheson KA. Predicting two-year longitudinal MD Anderson <u>Dysphagia</u> Inventory outcomes after intensity modulated radiotherapy for locoregionally advanced oropharyngeal carcinoma. Laryngoscope. 2017 Apr;127(4):842-848. doi: 10.1002/lary.26153. Epub 2016 Jul 21. PMID: 27440393; PMCID: PMC5253123.

108: Martin A, Murray L, Sethugavalar B, Buchan C, Williams GF, Sen M, Prestwich RJD. Changes in Patient-reported Swallow Function in the Long Term After Chemoradiotherapy for Oropharyngeal Carcinoma. Clin Oncol (R Coll Radiol). 2018 Dec;30(12):756-763. doi: 10.1016/j.clon.2018.06.013. Epub 2018 Jul 17. PMID: 30025978.

109: Pandey M, Karthikeyan S, Joshi D, Kumar M, Shukla M. Results of a randomized controlled trial of level IIb preserving neck dissection in clinically node-negative squamous carcinoma of the oral cavity. World J Surg Oncol. 2018 Nov 8;16(1):219. doi: 10.1186/s12957-018-1518-z. PMID: 30409212; PMCID: PMC6225726.

110: Prestwich RJ, Teo MT, Gilbert A, Williams G, Dyker KE, Sen M. Long-term swallow function after chemoradiotherapy for oropharyngeal cancer: the influence of a prophylactic gastrostomy or reactive nasogastric tube. Clin Oncol (R Coll Radiol). 2014 Feb;26(2):103-9. doi: 10.1016/j.clon.2013.10.005. Epub 2013 Nov 15. PMID: 24246275.

111: Samuels SE, Tao Y, Lyden T, Haxer M, Spector M, Malloy KM, Prince ME, Bradford CR, Worden FP, Schipper M, Eisbruch A. Comparisons of dysphagia and quality of life (QOL) in comparable patients with HPV-positive oropharyngeal cancer receiving chemo-irradiation or cetuximab-irradiation. Oral Oncol. 2016 Mar;54:68-74. doi: 10.1016/j.oraloncology.2015.12.001. Epub 2016 Jan 6. PMID: 26776757; PMCID: PMC4754145.

112: Barrios R, Tsakos G, García-Medina B, Martínez-Lara I, Bravo M. Oral health-related quality of life and malnutrition in patients treated for oral cancer. Support Care Cancer. 2014 Nov;22(11):2927-33. doi: 10.1007/s00520-014-2281-5. Epub 2014 May 22. PMID: 24848576; PMCID: PMC4183889.

113: Ryzek DF, Mantsopoulos K, Künzel J, Grundtner P, Zenk J, Iro H, Psychogios G. Early stage oropharyngeal carcinomas: comparing quality of life for different treatment modalities. Biomed Res Int. 2014;2014:421964. doi: 10.1155/2014/421964. Epub 2014 Feb 25. PMID: 24719863; PMCID: PMC3955642.

114: Thrasyvoulou G, Vlastarakos PV, Thrasyvoulou M, Sismanis A. Horizontal (vs. vertical) closure of the neo-pharynx is associated with superior postoperative swallowing after total laryngectomy. Ear Nose Throat J. 2018 Apr-May;97(4-5):E31-E35. doi: 10.1177/0145561318097004-502. PMID: 29940691.

115: Prestwich RJD, Murray LJ, Williams GF, Tease E, Taylor L, George C, Cardale K, Dyker KE, Murray P, Sen M, Ramasamy S. Impact of choice of feeding tubes on long-term swallow function following chemoradiotherapy for oropharyngeal carcinoma. Acta Oncol. 2019 Aug;58(8):1187-1196. doi: 10.1080/0284186X.2019.1609698. Epub 2019 Apr 29. PMID: 31032694.

116: Barringer DA, Hutcheson KA, Sturgis EM, Kies MS, Lewin JS. Effect of induction chemotherapy on speech and swallowing function in patients with oral tongue cancer. Head Neck. 2009 May;31(5):611-7. doi: 10.1002/hed.20989. PMID: 19107949; PMCID: PMC4079054.

117: Keereweer S, Kerrebijn JD, Al-Mamgani A, Sewnaik A, Baatenburg de Jong RJ, van Meerten E. Chemoradiation for advanced hypopharyngeal carcinoma: a retrospective study on efficacy, morbidity and quality of life. Eur Arch Otorhinolaryngol.

2012 Mar;269(3):939-46. doi: 10.1007/s00405-011-1694-8. Epub 2011 Jul 8. PMID: 21739092; PMCID: PMC3275723.

118: Gondivkar SM, Gadbail AR, Sarode SC, Gondivkar RS, Yuwanati M, Sarode GS, Patil S. Measurement properties of oral health related patient reported outcome measures in patients with oral cancer: A systematic review using COSMIN checklist. PLoS One. 2019 Jun 27;14(6):e0218833. doi: 10.1371/journal.pone.0218833. PMID: 31247007; PMCID: PMC6597073.

119: Mowry SE, Tang C, Sadeghi A, Wang MB. Standard chemoradiation versus intensity-modulated chemoradiation: a quality of life assessment in oropharyngeal cancer patients. Eur Arch Otorhinolaryngol. 2010 Jul;267(7):1111-6. doi: 10.1007/s00405-009-1183-5. Epub 2009 Dec 29. PMID: 20039175; PMCID: PMC2874032.

120: Speyer R, Heijnen BJ, Baijens LW, Vrijenhoef FH, Otters EF, Roodenburg N, Bogaardt HC. Quality of life in oncological patients with oropharyngeal dysphagia: validity and reliability of the Dutch version of the MD Anderson Dysphagia Inventory and the Deglutition Handicap Index. Dysphagia. 2011 Dec;26(4):407-14. doi: 10.1007/s00455-011-9327-3. Epub 2011 Jan 29. PMID: 21279522; PMCID: PMC3224721.

Supplemental data S2: Mapping of functions or symptoms repetitively mentioned in publications on functional outcome in HNC-patients to functional domains

Functional or symptom	**Functional domain**
Eating, drinking<br>Swallowing<br>Dysphagia<br>Feeding, Feeding tube, percutaneous endoscopic gastrostomy, PEG<br>Trism, mouth opening, chewing, dental status[1]<br>Xerostomia, sticky saliva, dry mouth, (hypo-)salivation[1]<br>Aspiration<br>Taste alteration (dysgeusia)<br>Normality of diet	Food intake
Laryngectomy<br>Respiration, respiratory<br>Dyspnea<br>Breath, breathing<br>Tracheo(s)tomy<br>Pharyngeal, laryngeal, tracheal stenosis<br>Pharyngeal, laryngeal, tracheal edema<br>Vocal chord paresis	Breathing
Speaking<br>Dysarthria<br>Neuropathy, paresis<br>Tongue mobility<br>Voice, Voice rehabilitation<br>Communication (verbal)<br>Hoarseness<br>Nasality, nasalization, velopharyngeal insufficiency, rhinolalia, rhinophonia	Speech
Pain<br>Pain medication	Pain
Depression<br>Emotion, emotional<br>Antidepressants<br>Well-being<br>Distress<br>Enjoy life	Mood
Neck	Shoulder-neck mobility

Shoulder
Neck lymphedema
Induration, fibrosis
Range of motion

[1]Despite considerable effort, we have not been able to code the functional domain saliva using external criteria with verbal rating scales in a meaningful sequence. This functional domain was assigned to the functional domain food intake.

Supplemental data S3: Adapted Head and Neck Cancer Functional Integrity Scale (HNC-FIT Scale)

**Functional Domain**	**Integrity Grad**					
	**0**	**1**	**2**	**3**	**4**	**Due to tumor / treatment**
Food intake	No oral feeding; only via gastrostomy tube	Gastrostomy tube needed; some oral feeding possible	No gastrostomy tube, oral diet, but only liquid/soft food	No gastrostomy tube, diet/ swallowing near normal	normal	
Respiration	Tracheostoma, needs blocked cannula	Tracheostoma, speech cannula/no cannula	No tracheostoma, breathing difficulties at rest	No tracheostoma, breathing difficulties only on exertion	normal	
Speech	Not possible, without phonation	Difficult to understand, no phone calls	Telephoning possible	Easy to understand, but pronunciation/ voice changed	normal	
Pain	Pain despite opiate therapy	Controlled with opiates	Regularly needs non-opioid analgesics	Needs analgesics from time to time	normal	
Mood	Suicidal thoughts	Very depressed despite antidepressants	with antidepressants overall normal mood, very depressed without	Occasionally depressed, no antidepressants	normal	
Neck & shoulder mobility[1]	Stiff neck and/or shoulder, hardly any movement possible	Can hair hardly comb, looking backwards in car not possible	Combing with problems, looking backwards in car difficult	Combing and looking backwards in car slightly restricted	normal	

[1]The worse result of neck mobility and shoulder mobility is counted.

The Head and Neck Cancer Functional Integrity Scale (HNC-FIT-scale) is completed by the clinician during the patient interview at oncology follow-up visits. It takes 1-2

minutes to complete the questionnaire. The HNC-FIT scale includes the functional domains of food intake, breathing, speech, pain, mood, and neck and shoulder mobility. Functional integrity is graded on a scale of 0 to 4, with 0 indicating complete loss of normal function (worst outcome) and 4 indicating functional integrity (best outcome).

Supplemental data S4: Assessment of face and content validity of the Head and Neck Cancer Functional Integrity Scale (HNC-FIT scales) via semi-structured expert interviews

Head and neck cancer (HNC) and its treatment can lead to various functional impairments. The "head and neck functional integrity scales" (HNC-FIT scale) is a clinical instrument for rapid physician-rated assessment of basic functional outcomes in HNC-patients during the initial diagnosis and oncological follow-up investigation. As part of the development and empirical validation of this clinical tool, we would appreciate your opinion as physician or therapist involved in the treatment of HNC-patients within the framework of a semi-structured interview. Please read the HNC-FIT scales and the instructions on how to complete it carefully. Then imagine you are completing this clinical instrument with a HNC-patient in the context of initial diagnosis or oncological follow-up investigation.

As part A of the semi-structured interview, we would like you to rate the entire HNC-FIT scales for its face-validity on a 5-point Likert scale from 1 (“very good”) to 5 (“not sufficient”) and to rate each functional domain on the same 5-point Likert scale via 8 questions for their content validity.

As part B of this semi-structured interview, we would like you to express concerns, additions, advantages and disadvantages of the entire HNC-FIT scales and for each functional domain. In addition, we would like you to express whether you considered any functional domains not important or irritating and whether you would like to include any previously missed functional domains.

Part A

1.) Please, rate the **entire** HNC-FIT scales. The individual functional items...

		**1**	**2**	**3**	**4**	**5**
a)	…record the most important functions impaired in HNC patients	☐	☐	☐	☐	☐

2.) Please, rate the functional domain "**food intake**". This functional domain...

		**1**	**2**	**3**	**4**	**5**
a)	…is formulated comprehensibly	☐	☐	☐	☐	☐
b)	…is based on objective external criteria	☐	☐	☐	☐	☐
c)	…distinguishes impaired from non-impaired HNC-patients	☐	☐	☐	☐	☐
d)	...probably responds well to a change in functional integrity	☐	☐	☐	☐	☐
e)	...probably achieves similar results if completed by different physicians	☐	☐	☐	☐	☐
f)	...probably achieves similar results if functional integrity remains unchanged	☐	☐	☐	☐	☐
g)	...represent a comprehensible increase from worst to best functional integrity	☐	☐	☐	☐	☐
h)	...represent an equidistant increase from worst to best functional integrity	☐	☐	☐	☐	☐

3.) Please, rate the functional domain "**breathing**". This functional domain...

		**1**	**2**	**3**	**4**	**5**
a)	…is formulated comprehensibly	☐	☐	☐	☐	☐

b)	…is based on objective external criteria	☐	☐	☐	☐	☐
c)	…distinguishes impaired from non-impaired HNC-patients	☐	☐	☐	☐	☐
d)	...probably responds well to a change in functional integrity	☐	☐	☐	☐	☐
e)	...probably achieves similar results if completed by different physicians	☐	☐	☐	☐	☐
f)	...probably achieves similar results if functional integrity remains unchanged	☐	☐	☐	☐	☐
g)	...represent a comprehensible increase from worst to best functional integrity	☐	☐	☐	☐	☐
h)	...represent an equidistant increase from worst to best functional integrity	☐	☐	☐	☐	☐

4.) Please, rate the functional domain "**speech**". This functional domain...

		**1**	**2**	**3**	**4**	**5**
a)	…is formulated comprehensibly	☐	☐	☐	☐	☐
b)	…is based on objective external criteria	☐	☐	☐	☐	☐
c)	…distinguishes impaired from non-impaired HNC-patients	☐	☐	☐	☐	☐
d)	...probably responds well to a change in functional integrity	☐	☐	☐	☐	☐
e)	...probably achieves similar results if completed by different physicians	☐	☐	☐	☐	☐
f)	...probably achieves similar results if functional integrity remains unchanged	☐	☐	☐	☐	☐
g)	...represent a comprehensible increase from worst to best functional integrity	☐	☐	☐	☐	☐
h)	...represent an equidistant increase from worst to best functional integrity	☐	☐	☐	☐	☐

5.) Please, rate the functional domain "**pain**". This functional domain...

		**1**	**2**	**3**	**4**	**5**
a)	…is formulated comprehensibly	☐	☐	☐	☐	☐
b)	…is based on objective external criteria	☐	☐	☐	☐	☐
c)	…distinguishes impaired from non-impaired HNC-patients	☐	☐	☐	☐	☐
d)	...probably responds well to a change in functional integrity	☐	☐	☐	☐	☐
e)	...probably achieves similar results if completed by different physicians	☐	☐	☐	☐	☐
f)	...probably achieves similar results if functional integrity remains unchanged	☐	☐	☐	☐	☐
g)	...represent a comprehensible increase from worst to best functional integrity	☐	☐	☐	☐	☐
h)	...represent an equidistant increase from worst to best functional integrity	☐	☐	☐	☐	☐

6.) Please, rate the functional domain "**mood**". This functional domain...

		1	2	3	4	5
a)	…is formulated comprehensibly	☐	☐	☐	☐	☐
b)	…is based on objective external criteria	☐	☐	☐	☐	☐
c)	…distinguishes impaired from non-impaired HNC-patients	☐	☐	☐	☐	☐
d)	...probably responds well to a change in functional integrity	☐	☐	☐	☐	☐
e)	...probably achieves similar results if completed by different physicians	☐	☐	☐	☐	☐
f)	...probably achieves similar results if functional integrity remains unchanged	☐	☐	☐	☐	☐
g)	...represent a comprehensible increase from worst to best functional integrity	☐	☐	☐	☐	☐
h)	...represent an equidistant increase from worst to best functional integrity	☐	☐	☐	☐	☐

7.) Please, rate the functional domain "**<u>shoulder-neck mobility</u>**". This functional domain...

		1	2	3	4	5
a)	…is formulated comprehensibly	☐	☐	☐	☐	☐
b)	…is based on objective external criteria	☐	☐	☐	☐	☐
c)	…distinguishes impaired from non-impaired HNC-patients	☐	☐	☐	☐	☐
d)	...probably responds well to a change in functional integrity	☐	☐	☐	☐	☐
e)	...probably achieves similar results if completed by different physicians	☐	☐	☐	☐	☐
f)	...probably achieves similar results if functional integrity remains unchanged	☐	☐	☐	☐	☐
g)	...represent a comprehensible increase from worst to best functional integrity	☐	☐	☐	☐	☐
h)	...represent an equidistant increase from worst to best functional integrity	☐	☐	☐	☐	☐

Part B

1.) Please, express concerns, additions, advantages and disadvantages for the **entire** HNC-FIT scale:

____
____
____
____
____

2.) Please, express concerns, additions, advantages and disadvantages for the functional domain **“food intake”**:

a)____
b)____
c)____
d)____
e)____

f)____
g)____
h)____

3.) Please, express concerns, additions, advantages and disadvantages for the functional domain **<u>“breathing”</u>**:
a)____
b)____
c)____
d)____
e)____
f)____
g)____
h)____

4.) Please, express concerns, additions, advantages and disadvantages for the functional domain **<u>“speech”</u>**:
a)____
b)____
c)____
d)____
e)____
f)____
g)____
h)____

5.) Please, express concerns, additions, advantages and disadvantages for the functional domain **<u>“pain”</u>**:
a)____
b)____
c)____
d)____
e)____
f)____
g)____
h)____

6.) Please, express concerns, additions, advantages and disadvantages for the functional domain **<u>“mood”</u>**:
a)____
b)____
c)____
d)____
e)____
f)____
g)____
h)____

7.) Please, express concerns, additions, advantages and disadvantages for the functional domain **<u>“shoulder-neck mobility”</u>**:
a)____
b)____
c)____
d)____
e)____

f)____
g)____
h)____

8.) Related to the HNC-FIT scales, were there any functional domains that you considered **not** important?
Functional domain “food intake”, because:

Functional domain “breathing”, because:

Functional domain “speech”, because:

Functional domain “pain”, because:

Functional domain “mood” because:

Functional domain “shoulder-neck mobility”, because:

9.) Related to the HNC-FIT scale, were there any functional domains that you found irritating?
Functional domain “food intake”, because:

Functional domain “breathing”, because:

Functional domain “speech”, because:

Functional domain “pain”, because:

Functional domain “mood” because:

Functional domain “shoulder-neck mobility”, because:

10.) Related to the HNC-FIT scales, where there any additional functional domains that should be included?

Thank you for your participation!

Supplemental data S5: Instructions for clinicians on how to complete the Head and Neck Functional Integrity Scale.

The functional domain food intake includes eating and drinking. ‘Unable to swallow; only via gastrostomy tube’ means that the patient is unable to swallow, and his intake of food is exclusively via gastrostomy (or nasogastral) tube. This applies also for nasogastric tubes. ‘Gastrostomy tube needed, some oral feeding possible’ means that the patient relies on PEG for adequate food and fluid intake, but occasional sips are possible. ‘No gastrostomy tube, oral diet, but only liquid/soft food’ means that the patient does not have a gastrostomy tube but cannot eat solid food. ‘No gastrostomy tube, diet near normal’ means that the patient can essentially eat normally, but with certain (e.g. dry foods) problems, but these can be overcome (for example, by simultaneously taking liquid). ‘Normal’, like in all functional domains, means that the function is as it was before the illness.

In the functional domain respiration 'Tracheostoma, needs blocked cannula' means that the patient has a tracheostoma and must use a cannula with blocked cuff, e.g. because of aspiration. ‘Tracheostoma, speech cannula/no cannula’ means a simple condition with tracheostomy. ‘No tracheostoma, breathing difficult at rest’ is ticked at dyspnea at rest. With ‘No tracheostoma, breathing difficulties only on exertion’, a typical loading situation would be e.g. climb stairs.

In the functional domain speech ‘Not possible, without phonation’ means that the patient is incapable of speech and essentially must rely on written communication. At 'Difficult to understand, no phone calls', the patient can indeed make sounds and you can understand the meaning with high concentration, but the patient in not able to make calls. At 'Telephoning possible' is the language is very limited and difficult to understand, but it is a communication over the phone with strangers possible. ‘Easy to understand, but pronunciation/voice changed’ also detects slight functional limitations of speech, but the communication is possible without any problems.

In the pain dimension ‘pain despite of opiate therapy'' means that the patient suffers from pain despite pain therapy with opiates by experienced pain specialists. 'Controlled with opiates' means that with adequate pain therapy including opiates the patient is essentially painless. 'Regularly needs non-opioid analgesics' means that the patient has a long-term pain therapy without opioids and is thus essentially painless. 'Needs analgesics from time to time' means occasional pain and occasionally use of painkillers.

In the dimension mood, 'suicidal thoughts' means that the patient is so depressed that he has suicidal thoughts. As a rule, psychiatric intervention is urgently needed. 'Very depressed despite antidepressants' means that the patient has sustained depressive mood despite adequate antidepressant therapy received by a suitable medical facility. 'With antidepressants overall normal mood' means that the patient regularly needs antidepressants because of depressive mood. With this antidepressant therapy, however, the mood is essentially normal. 'Occasionally depressed, no antidepressants needed' is ticked in case of occasional depressive mood.

In the shoulder-neck-mobility dimension two functions are queried simultaneously, namely shoulder mobility and neck mobility. In each case the worse functional status is ticked. ‘Stiff neck and/or shoulder, hardly any movement possible'' is a complete fixation in the neck and/or shoulder area. 'Can hair hardly comb, looking backwards in car not possible' means that the shoulder mobility is so severely limited that with a straight head not all areas of the head are reachable with the comb and/or that without a rear view camera the car cannot be reversed under visual control because the head cannot be turned far enough. 'Combing with problems, looking backwards in car difficult' means that combing the hair when the head is straight and that reversing under sight is possible, but means a considerable effort. 'Combing and looking backwards in car near normal' means

a slight restriction of the neck and shoulder mobility without significant functional impairment.

Supplemental data S6: Selection of HNC-related symptoms and functions not covered by HNC-FIT scales.

- Sight
- Hearing, Tinnitus
- Hyposmia, smell disturbances
- Balance, vertigo
- Neuropathies, sensation, proprioception

- Sleep, Sleep disturbances
- Fatigue
- Thyroid dysfunction

- Body image
- Sexuality
- Disfigurement

- Cognitive function
- Anxiety and concerns

- Appetite
- Nausea
- Weight loss
- Mucositis/stomatitis

- Dermatitis
- Hair loss/alopecia
- Hyperpigmentation or hypopigmentation
- Soft-tissue- osteonecrosis
- Chronic wounds, ulcers

- Social function, doing s.th. in public
- Work status
- Performance status (ECOG/Karnofsky)
- Cough

Supplemental data S7: Mean scores and standard deviations of the adapted HNC-FIT-scales.

Functional domain	Controls	Pretreatment group	Posttreatment group
Food intake	3.9+/-0.8	3.6+/-1.3	2.8+/-0.7
Breathing	3.9+/-0.6	3.7+/-1.2	3.1+/-0.7
Speech	3.9+/-0.6	3.7+/-1.2	3.1+/-0.7
Pain	3.9+/-0.8	3.6+/-1.2	3.4+/-0.9
Mood	3.7+/-0.8	3.4+/-0.8	3.4+/-0.9
Mobility	3.9+/-0.5	3.9+/-0.9	3.5+/-0.3

Supplemental data S8: German Version of the adapted Head and Neck Cancer Functional Integrity Scale (HNC-FIT Scale)

**Funktions Domäne**	**Grad der Integrität**					
	**0**	**1**	**2**	**3**	**4**	
Ernährung	Keine orale Narhungsaufnahme; nur über	Magensonde nötig; orale Ernährung teilweise möglich	Keine Magensonde, orale Ernährung nur flüssig/breiig/passiert	Keine Magensonde, Ernährung/Schlucken annähernd normal	Normal	**Durch Tumorerkrankung / - behandlung**
Atmung	Tracheostoma, bracuth geblockte Kanüle	Tracheostoma, Sprechkanüle/keine Kanüle	Kein Tracheostoma, Atmung in Ruhe ershwert	Kein Tracheostoma, Atmung bei Belastung ershwert	Normal	
Sprechen	Nicht möglich, ohne Phonation	Schwer verständlich, telefonieren nicht möglich	Telefonieren möglich	Gut verständlich, aber Aussprache oder Stimme verändert	Normal	
Schmerzne	Schmerzen trotz Opiattherapie	Kontrolliert mit Opiaten	Braucht regelmäßig Nicht-Opioid-Analgetika	Bracuht ab und zu Analgetika	Normal	
Stimmung	Suizidgedanken	Auch mit Antidepressiva sehr niedergedrückt	Mit Andipressiva insgesamt normale Stimmung, ohne	Gelegentlich gedrückte Stimmung, keine Antidepressiva nötig	Normal	
Schulter-Hals-Beweglichkeit[1]	Hals steif, kaum Bewegung möglich	Haare kämmen kaum, umsehen im Auto nicht möglich	Haare kämmen mit Problemen, Umsehen im Auto gerade eben	Etwas eingeschränkt, haare kämmen problemlos möglich, Umsehen im Auto kein Problem	Normal	

[1]The worse result of neck mobility and shoulder mobility is counted.
